# Constitutive expression of CD26/dipeptidylpeptidase IV on peripheral blood B lymphocytes of patients with B chronic lymphocytic leukaemia

**DOI:** 10.1038/sj.bjc.6690167

**Published:** 1999-03

**Authors:** B Bauvois, I De Meester, J Dumont, D Rouillard, H X Zhao, E Bosmans

**Affiliations:** Unité 365 INSERM, Institut Curie, Paris, France; Section de Recherche, Institut Curie, Paris, France; Department of Pharmaceutical Sciences, University of Antwerp, Wilrijk, Belgium; Service Hématologie Clinique, Institut Curie, Paris, France; Eurogenetics Headquarters, Tessenderlo, Belgium

**Keywords:** dipeptidylpeptidase IV, CD26 antigen, B-CLL, B-cell activation

## Abstract

We have investigated the expression of the ectoenzyme dipeptidylpeptidase IV (DPP IV)/CD26 on lymphocytes obtained from patients with B chronic lymphocytic leukaemia (B-CLL) and compared it with healthy subjects. Using two-colour immunofluorescence analysis with CD26 and CD20 or CD23 monoclonal antibodies, CD26 was found undetectable on peripheral resting B-cells (CD20^+^ CD23^−^) from normal donors whereas it was expressed on B-cells activated in vitro with interleukin (IL)-4 and *Staphylococcus aureus* strain *cowan I* (CD20^+^ CD23^+^). The expression of CD26 on leukaemic B-cells (CD20^+^ CD23^+^) was clearly induced in 22 out of 25 patients examined. Consequently, induced levels of CD26 cell surface expression on either normal activated and malignant B-cells coincided with the enhancement of DPP IV activity detected on the surface of these cells. Reverse transcription polymerase chain reaction analyses showed that the transcript levels of the CD26 gene was higher in normal activated B-cells and B-CLL cells than in resting B-cells, suggesting that CD26 was expressed at the level of transcriptional activation. These observations provide evidence of the abnormal expression of DPPIV/CD26 in B-CLL which, therefore, may be considered as a novel marker for B-CLL. Further investigation in relation to CD26 expression and other B malignancies needs to be defined. © 1999 Cancer Research Campaign


British Journal of Cancer (1999) 79(7/8), 1042–1048
© 1999 Cancer Research Campaign
bjoc.1998.0167

### Constitutive expression of CD26/dipeptidylpeptidase IV on peripheral blood B lymphocytes of patients with B chronic lymphocytic leukaemia

**B Bauvois[1], I De Meester[3], J Dumont[4], D Rouillard[2], HX Zhao[1] and E Bosmans[5]**

[1]Unité 365 INSERM, Institut Curie, 75231 Paris cedex 05, France; [2]Institut Curie, Section de Recherche, 75231 Paris cedex 05, France; [3]Department of Pharmaceutical Sciences, University of Antwerp, B-2610, Wilrijk, Belgium; [4]Service Hématologie Clinique, Institut Curie, 75231 Paris cedex 05, France; [5]Eurogenetics Headquarters, B-3980, Tessenderlo, Belgium

**Summary** We have investigated the expression of the ectoenzyme dipeptidylpeptidase IV (DPP IV)/CD26 on lymphocytes obtained from patients with B chronic lymphocytic leukaemia (B-CLL) and compared it with healthy subjects. Using two-colour immunofluorescence analysis with CD26 and CD20 or CD23 monoclonal antibodies, CD26 was found undetectable on peripheral resting B-cells ($CD20^+$ $CD23^-$) from normal donors whereas it was expressed on B-cells activated in vitro with interleukin (IL)-4 and *Staphylococcus aureus* strain *cowan I* ($CD20^+$ $CD23^+$). The expression of CD26 on leukaemic B-cells ($CD20^+$ $CD23^+$) was clearly induced in 22 out of 25 patients examined. Consequently, induced levels of CD26 cell surface expression on either normal activated and malignant B-cells coincided with the enhancement of DPP IV activity detected on the surface of these cells. Reverse transcription polymerase chain reaction analyses showed that the transcript levels of the CD26 gene was higher in normal activated B-cells and B-CLL cells than in resting B-cells, suggesting that CD26 was expressed at the level of transcriptional activation. These observations provide evidence of the abnormal expression of DPPIV/CD26 in B-CLL which, therefore, may be considered as a novel marker for B-CLL. Further investigation in relation to CD26 expression and other B malignancies needs to be defined.

**Keywords:** dipeptidylpeptidase IV; CD26 antigen; B-CLL; B-cell activation

Cell surface-associated proteases are involved in many physiological processes ranging from morphogenesis and tissue differentiation to the general regulation of haemostasis. Apart from their role in terminal degradation of proteins and regulation of hormone levels, some of these enzymes appear to be functionally involved in cell activation (Fleisher, 1994; Bauvois, 1997; Letarte et al, 1997). Of these ectoenzymes, the serine ectoprotease dipeptidylpeptidase IV (DPP IV) (EC 3.4.14.5) identical to CD26 antigen, has been shown to be a marker for T lymphocyte activation (Fleisher, 1994). CD26/DPP IV is associated with the protein tyrosine phosphatase CD45, and at the level of its extracellular domain with adenosine deaminase-1 (ADA-1), the lack of which is associated with severe combined immunodeficiency (Fleisher, 1994). CD26/DPP IV is abundantly expressed in kidney, lung, small intestine and liver (Fleisher, 1994). In the immune system, CD26/DPP IV is found primarily at low density on subsets of $CD4^+$ and $CD8^+$ T-cells and on $CD3^+$ medullary thymocytes (Fleisher, 1994) and on activated natural killer (NK) and B-cells (Bühling et al, 1994; Fleisher 1994).

Enzyme cytochemical and/or immunophenotypic approaches of CD26/DPP IV suggested this enzyme to be a useful marker in some pathologies. Overexpression of CD26/DPP IV was observed in differentiated thyroid carcinomas (Tanaka et al, 1995) and on T blood cells from patients with autoimmune diseases including progressive multiple sclerosis, Graves' disease and rheumatoid arthritis (Hafler et al, 1985; Eguchi et al, 1989; Nakao et al, 1989). In contrast, the loss of CD26/DPP IV molecule from the surface of T-cells has been associated with T-CLL (chronic lymphocytic leukaemia) and ALL (acute lymphocytic leukaemia) diseases (Kondo et al, 1996), oral cancer (Uematsu et al, 1996) and HIV infection (Vanham et al, 1993). CD26/DPP IV expression in human lymphomas appeared to be restricted to $CD30^+$ anaplastic large cells (irrespective of their T/B phenotype) and to a subset of non-Hodgkin's T-cells whereas non-Hodgkin's B-cells did not express CD26 antigen (Carbone et al, 1994). Conflicting data were, however, reported with the detection of a high DPP IV activity in B-cell lysates from patients with non-Hodgkin's lymphoma when compared with those from normal individuals (Khalaf et al, 1987). Moreover, attempts have been made to correlate the expression of DPP IV with B-CLL, with controversial results. Indeed, some studies demonstrated the presence of low but detectable levels of DPP IV activity in lysates of B-CLL cells (Feller et al, 1983; Srivastava and Bhargava, 1986; Scott et al, 1988) or on intact B-CLL cells (Bylinka et al, 1992). In contrast, two cytochemical studies indicated that B-CLL cells failed to express a DPP IV activity (Andrews et al, 1985; Invernizzi et al, 1985).

The objective of this study was to assess the expression of CD26/DPP IV molecules on the surface of lymphocytes of B-CLL patients in comparison with that of healthy subjects. We show here a clear correlation between B-CLL malignancy and CD26/DPP IV cell surface expression that might be implicated in the regulation of lymphocyte proliferation.

*Received 7 May 1998*
*Revised 4 August 1998*
*Accepted 5 August 1998*

*Correspondence to:* B Bauvois, Unité 365 INSERM, Institut Curie, Pavillon Pasteur, 26 rue d'Ulm, 75231 Paris cedex 05, France

### MATERIALS AND METHODS

### Antibodies and reagents

Rhodormine (RD)-conjugated CD26 (Ta1, mIgG1) monoclonal antibody (mAb), RD-conjugated mouse $IgG_1$ ($mIgG_1$), fluorescein isothiocyanate (FITC)-conjugated $mIgG_1$ and $mIgG_{2a}$ were obtained from Coulter (USA). FITC-conjugated –CD20 (B9E9, $mIgG_{2a}$), –CD3 (×35, $mIgG_{2a}$), –CD23 (9P.25, $mIgG_1$), –CD5 (BL1a, $mIgG_{2a}$) and FITC-conjugated CD14 (RM052, $mIgG_{2a}$) were from Immunotech (Marseille, France). Magnetic beads coated with $CD19^+$ were purchased from Dynal (Oslo, Norway). TA5.9 mAb directed against CD26 ($mIgG_1$) was characterized previously (De Meester et al, 1993). Ficoll-Hypaque was purchased from Pharmacia (Uppsala, Sweden). *Staphylococcus aureus* strain *cowan I* (SAC) was obtained from Clini-Sciences (Montrouge, France). Recombinant human interleukin-4 (rIL-4) was purchased from Immugenex (Los Angeles, CA, USA; sp. act.: $10^8$ U $mg^{-1}$). Gly-Pro-p-nitroanilide (pNA) was purchased from Sigma Chemical Co. (St Louis, MO, USA).

### Cells

Human mononuclear cells were isolated from heparinized normal peripheral blood by Ficoll-Hypaque (Pharmacia) density gradient centrifugation. B-cells were isolated using beads coated with Abs to CD19 and detached with Detachabeads (Dynal) and the B-enriched population contained ≤ 5% $CD3^+$ and ≤ 5% $CD14^+$ cells. B-cell cultures were performed in flat-bottomed 24-well microtiter with each well containing purified B-cells ($1 \times 10^6$) in 1 ml of Roswell Park Memorial Institute (RPMI)-1640 medium plus 5% fetal calf serum (FCS), containing 2 mM glutamine and 10 μg $ml^{-1}$ gentamycin. Some cell cultures were stimulated with rIL-4 (100 U $ml^{-1}$) or SAC (0.01%) or combinations of rIL-4 plus SAC, at 37°C for 2 days in a humidified atmosphere containing 5% carbon dioxide. Untreated patients (25) with B-CLL (17 males and eight females) were included in the study. B-CLL diagnosis was established according to the international CLL workshop criteria including peripheral blood lymphocyte morphology and coexpression of CD5, CD20 and CD23 antigens.

### Flow cytometry analysis

Cells were immunostained as previously described (Bauvois et al, 1996). Analysis was performed on a FACS flow cytometer analyser (Becton-Dickinson, Mountain View, CA, USA); 10 000 events were recorded and analysed using the Lysys software (Becton Dickinson). Fluorescence data were expressed in relative fluorescence intensity (%) and antigen relative density per cell was obtained by subtracting the peak channel number of the negative control from the peak channel number of the corresponding experimental sample.

### DPP IV activity assay

DPP IV activity at the surface of intact cells was measured spectrophotometrically by hydrolysis of Gly-Pro-pNA and formation of pNA, as previously described (Bauvois et al, 1992).

### RNA extraction and RT-PCR

Total RNA was prepared by acid guanidinium thiocyanate–phenol–chloroform extraction from cells (Chomeczynski and Sacchi, 1987). A 0.5 μg total RNA was reverse transcribed with 0.2 μg random hexamers (Boehringer, Vienna, Austria) in the presence of 200 U Moloney murine leukaemia reverse trancriptase (RT; BRL, Meylan, France). CD26 cDNA was amplified using the sense primer 5′-ATG GAC GGG GAA AGA AGA TA-3′ corresponding to bases 568–587 of the human cDNA sequence (Abbott et al, 1994) and the antisense primer 5′-TTT ACA GTT GGA TTC ACA GCT C-3′ corresponding to bases 789–810, to generate a 223-bp product. β2-microglobulin cDNA was amplified using the sense primer 5′- CAT CCA GCG TAC TCC AAA GA-3′ and antisense 5′-GAC AAG TCT GAA TGC TCC AC-3′ to generate a 165-bp product. The polymerase chain reaction (PCR) mixture comprised 50 ng template cDNA, 67 mM Tris-HCl (pH 8.8), 16.6 mM ammonium sulphate ($(NH_4)_2SO_4$), 2.7 mM magnesium chloride, 1 mM dNTP, 0.5 μM of each primer and 1.25 U *Taq* polymerase (Boehringer, Vienna, Austria) in a total volume of 100 μl. Reactions were performed in DNA thermal cycler (Perkin Elmer) with 35 cycles of 94°C for 50 s (denaturation), 57°C for 50 s (annealing) and 72°C for 20 s (elongation). In all experiments, a free blank was tested as a check for contamination. The PCR products were first visualized by electrophoresis in 2% agarose gel containing 0.2 μg $ml^{-1}$ ethidium bromide. For final detection, the PCR products were separated on 7% polyacrylamide gels and visualized using ethidium bromide.

### RESULTS AND DISCUSSION

### Differential expression of CD26 molecules on the surface of B-CLL and control group (resting and activated) B-cells

By using one-colour FACS analysis, it was previously suggested that a small subset (≤ 5%) of normal peripheral B-cells reacted with the mAb Ta1 raised against the CD26 molecule from activated T-cells (Bühling et al, 1994). In this study, two-colour FACS analysis was used to determine the levels of Ta1/CD26 expression on $CD20^+$ B-cells. We identified on peripheral blood lymphocytes (PBL) cells from normal healthy donors, a $CD20^+$ B-cell population negative for Ta1/CD26 (Figure 1.3). Following cell isolation, purified $CD20^+$ B-cells were unreactive with CD26/Ta1 mAb (Figure 1.6) and with CD23 mAb (data not shown). As illustrated in Figure 2, isolated $CD20^+$ $CD23^-$ B-cells from seven healthy donors were found $CD26^-$. Bühling et al (1994) previously found that SAC increased CD26 expression on B-cells. Moreover, the physiological B-cell stimulatory factor IL-4 is known to increase the expression of a variety of molecules on B-cells including CD23 (Kolb et al, 1991). Thus, resting $CD20^+$ $CD23^-$ $CD26^-$ B cells were in vitro cultured for 2 days in the absence or in the presence of rIL-4 or SAC, or combinations of rIL-4 and SAC. Our study confirmed the enhancing effect of SAC and revealed new findings with regard to its association with IL-4. When co-stained with CD23 and CD26/Ta1 mAbs, most of rIL-4-stimulated B-cells showed expression of CD23 antigen (Figure 3.6) whereas SAC-stimulated B-cells showed increase in CD26 expression (Figure 3.9). Combination of rIL-4 and SAC stimulation resulted in two subpopulations of activated B-cells, one subpopulation $CD23^+$ $CD26^-$ and the other $CD23^+$ $CD26^+$ (Figure 3.12). Analysis of the results obtained from four separate experiments indicate that 20–50% activated B-cells were $CD23^+$ $CD26^+$ (Figure 2).

When CD26 expression was investigated in 25 cases of B-CLL (positive for CD5, CD20 and CD23), three cases were found negative for CD26/Ta1 whereas 22 cases displayed a very moderate to

© Cancer Research Campaign 1999

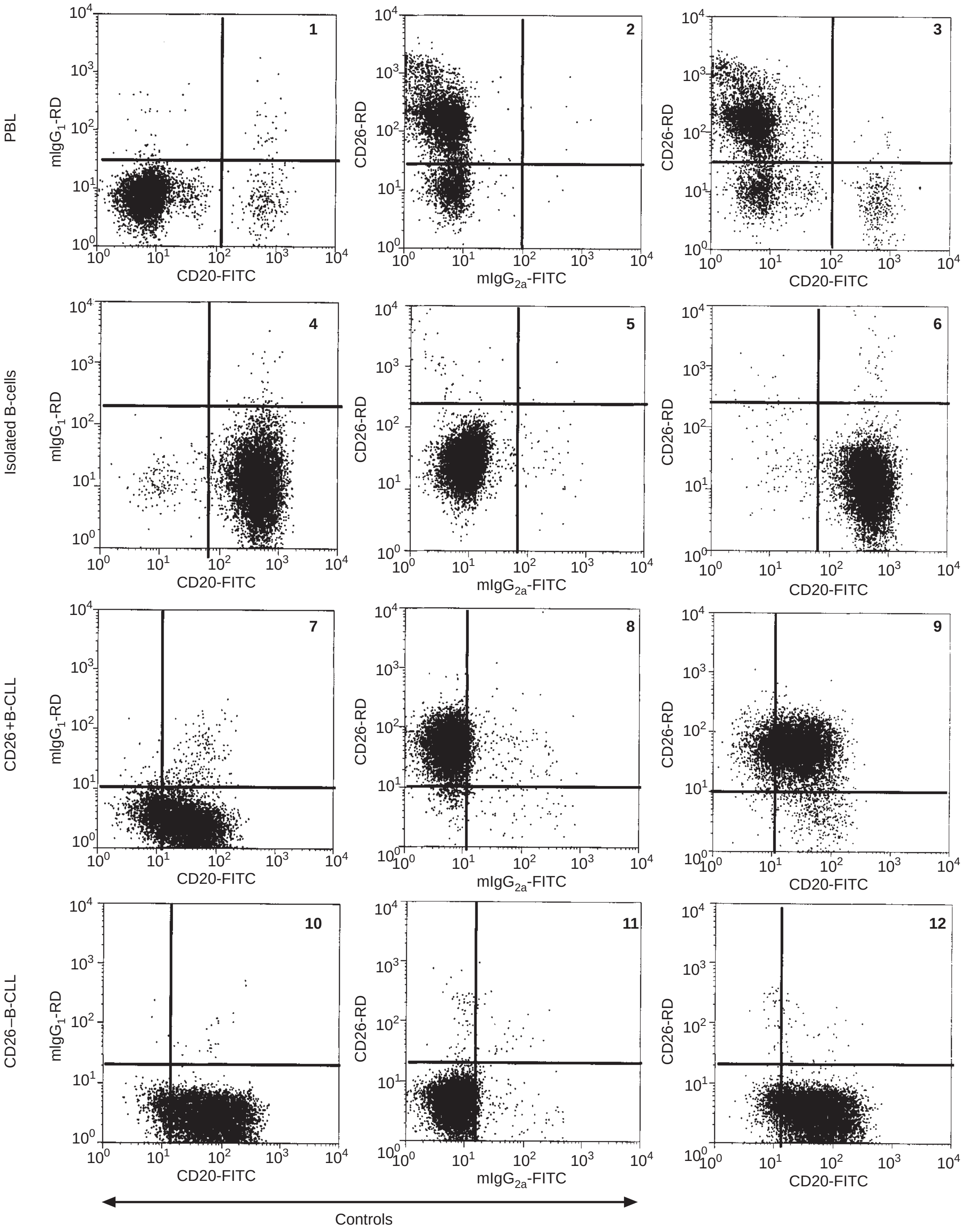

**Figure 1** Representative cytograms of normal B and leukaemic B-CLL cells costained with CD26 and CD20 mAbs. PBMC cells and isolated B-cells from one normal donor, and B-cells from two B-CLL patients obtained as described in Material and methods, were examined by two-colour immunofluorescence staining in flow cytometry analysis. Cells were stained with CD20-FITC/mIgG$_1$-RD (1, 4, 7, 10) or mIgG$_{2a}$-FITC/Ta1-RD (2, 5, 8, 11) or CD20-FITC/Ta1-RD (3, 6, 9, 12). The *x*-axis shows log green fluorescence and the *y*-axis shows red fluorescence. Quadrants delineated by squares indicate negative and positive populations of cells as determined using negative controls (1 and 2, 4 and 5, 7 and 8, 10 and 11)

*© Cancer Research Campaign 1999*

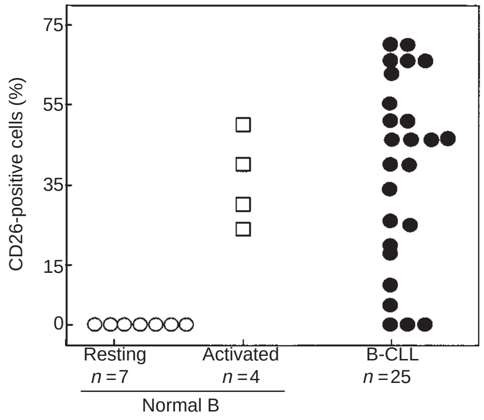

**Figure 2** Analysis of CD26 expression on B-CLL cells and normal counterpart B cells. Cells were assessed for CD26 expression using Ta1 mAb. B-CLL cells ● ($n$ = 25); normal resting B-cells ○ ($n$ = 7); normal B-cells activated in vitro with rIL-4/SAC □ ($n$ = 4). CD26 expression was determined as the percentage of positive cells strong staining of cell surface CD26 (from 5 to 70% positive cells, Figure 2). Representative positive- and negative-CD26 FACS cytograms of cells from two patients, stained with CD20 and CD26 mAbs, are presented in Figure 1.9 and 1.12, respectively. It was noted that CD26/TA5.9 mAb similarly detected CD26 antigens on activated B-cells (data not shown).

To determine whether the differences observed in cell surface CD26 expression from normal (activated vs resting) and leukaemic B-cells, could be accounted for by the abundance of CD26 mRNA, RT-PCR experiments were carried out on total mRNA extracted from various B-cell populations. As shown in Figure 4, samples were standardized for total cDNA content by assessing the presence of identical amounts of β2-microglobulin transcript. As controls, the expected 223-bp CD26 product was observed in activated T-cells and human dermal fibroblasts, respectively (Figure 4, lanes 12 and 13). As shown in Figure 4, the 223-bp-specific band was detected at very low levels in resting $CD26^-$ B-cells from three donors (lanes 1–3) whereas an increased CD26 signal was found in $CD23^+$-activated B-cells (lane 4 compared to lane 3) and in five B-CLL samples (lanes 6, 8–11) positive for CD26 cell surface expression. Thus, it appears that CD26 mRNA was increased in $CD23^+$ B-cells and that this increase was followed by an induction of CD26 molecules at the surface of these cells.

### Cell surface CD26 up-regulation is correlated with an increase in cell surface DPP IV activity

The CD26 molecule is identical to the serine ectoprotease DPP IV (Fleisher, 1994). DPP IV activity is usually assessed by measuring the rate of hydrolysis of the chromogenic substrate Gly-Pro-pNA and inhibition of the cleavage by specific inhibitors of DPP IV activity (DFP, diprotin A) (Bauvois et al, 1992). We therefore investigated Gly-Pro-pNA hydrolysis by normal resting and activated B-cells and B-CLL cells. With resting B-cells, a basal activity was detected (< 200 pmole/30 min/$10^5$ cells, Figure 5). After IL-4/SAC treatment, DPP IV activity was markedly increased by a factor of 2–3 and this stimulation paralleled the increase in CD26 expression we observed under the same conditions (Figure 5). When 17 B-CLL cells were co-analysed for CD26 expression and DPP IV activity, a significant correlation was also found between DPP IV activity and CD26 expression determined as the per cent of positive cells (Figure 5). A similar correlation was obtained between DPP IV activity and the number of CD26 molecules displayed by individual cells (relative amount of antigen density per cell, data not shown). Together, these results suggest that up-regulated levels of DPP IV activity on $CD23^+$ cells were attributable to the induction of CD26.

### CONCLUSION

In this study, we have investigated CD26/DPP IV expression in B lymphocytes from normal adults and compared with their neoplastic counterpart B-cells from patients with B-CLL. B-CLL represents a chronic lymphoproliferative disorder with a high biological and clinical heterogeneity. We found that normal peripheral $CD23^-$ B-cells from healthy donors are negative for cell surface CD26. When B lymphocytes become in vitro activated with IL-4 and SAC, the expression of CD26 was induced on $CD23^+$ B-cells. Our major finding concerns the constitutive expression of CD26 on $CD23^+$ cells from a majority of untreated patients with B-CLL (88%). RT-PCR analyses suggest that CD26 up-regulation was at the level of transcription activation. Recently, two additional forms of DPP IV have been characterized on T lineage cells (Duke-Cohan et al, 1996; Jacotot et al, 1996); both expressed DPP IV activity but were incapable of binding ADA-1 (Duke-Cohan et al, 1996; Jacotot et al, 1996). Additionally, a DPP IV protein closely related to DPP IV but without DPP IV activity has also been described (Yokotani et al, 1993). In our study, we found that the degree of Ta1/CD26 expression was in good correlation with the levels of DPP IV enzymatic activity, and that DPP IV activity was indeed attributable to the CD26 form endowed with the capacity to bind ADA (data not shown). Thus, our data suggest that malignant B-cells, like activated normal B-cells, express at their surface a CD26/DPP IV molecule similar to the major form of CD26/DPP IV present on activated T-cells. In vitro treatment of activated T- and B-cells with specific DPP IV inhibitors led to cell growth suppression and a decrease in cytokine or IgM production (Schön et al, 1987; Flentke et al, 1991; Bühling et al, 1994). A specific inhibitor of DPP IV prodipine has recently been shown to abrogate in vivo systemic IgM allo-Ab responses in rats (De Meester et al, 1997). With regard to T-cells, the 105 kDa CD26 molecule has a direct stimulatory function for T-cell proliferation and is able to transduce strong comitogenic signals through the same signal transduction pathways involved in T-cell activation via the CD3 complex (Fleisher, 1994; Hegen et al, 1997). Alternatively, it cannot be excluded that CD26/DPP IV, based on its enzymatic properties, functions to regulate cell growth through the activation/inactivation of crucial factors (Fleisher, 1994). Finally, with regard to its binding capacity to extracellular matrix components including collagens and fibronectin (Fleisher, 1994), CD26/DPP IV could enhance the invasive properties of tumour cells from bone into blood. Further investigation is therefore required to clarify the role, if any, of B-cell CD26 in the control of cell proliferation in leukaemia.

Concomitantly with the overexpression of CD26/DPP IV on B-CLL cells, we have recently observed significant higher levels of DPP IV activity (up to threefold) in sera from B-CLL patients as compared to normal sera (B Bauvois, unpublished results). Although DPP IV activity in serum may be released from various types of cells (Fleisher, 1994; Duke-Cohan et al, 1996),

© Cancer Research Campaign 1999

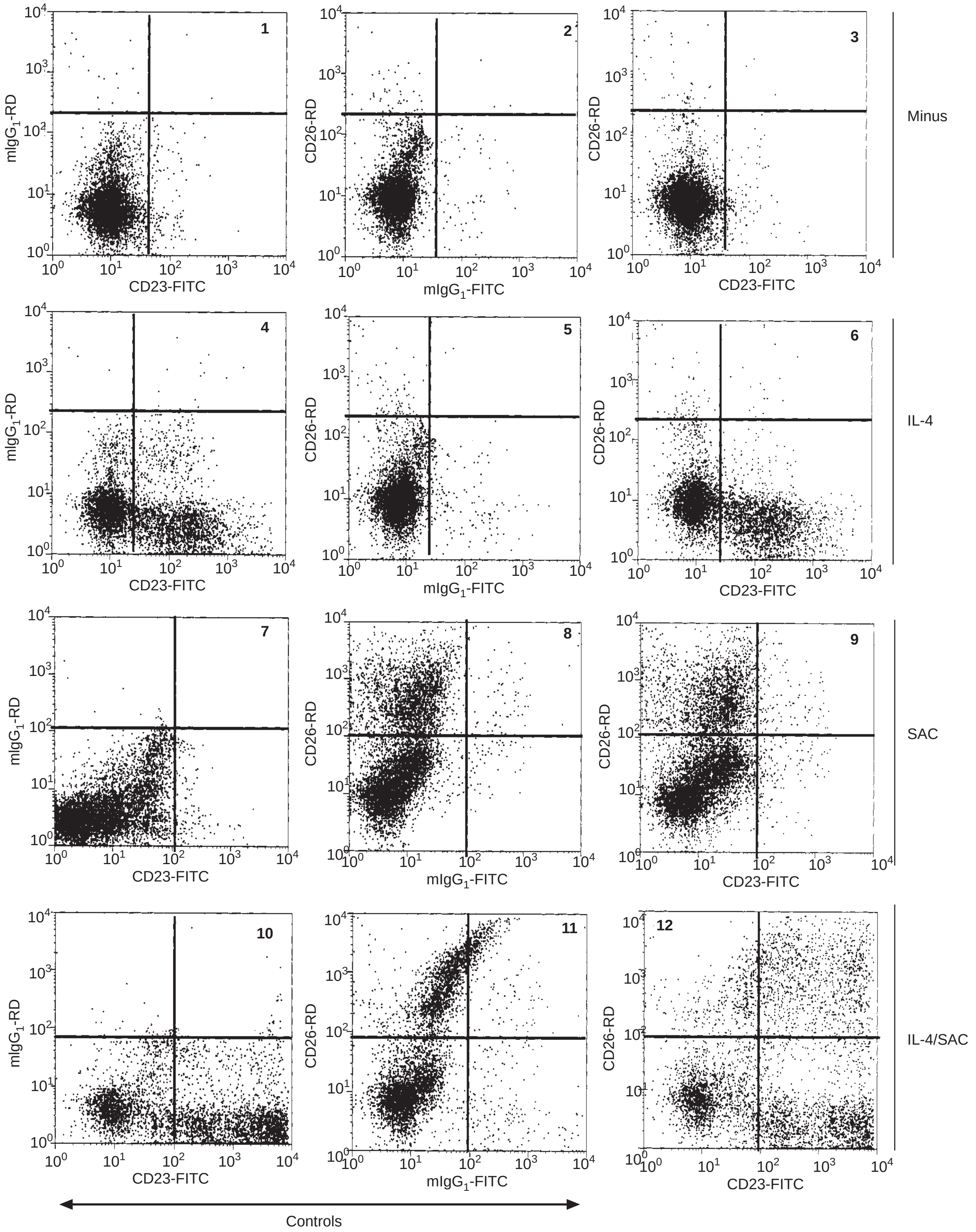

**Figure 3** Representative cytograms of activated B-cells costained with CD23 and CD26/Ta1 mAbs. $CD19^+$-isolated B-cells were cultured in the absence (1–3) or in the presence (4–6) of 100 U ml rIL-4, or 0.01% SAC (7–9) or combinations of SAC and rIL-4 (10–12) for 2 days. Cells were stained with CD23-FITC/ $mIgG_1$-RD (1, 4, 7, 10) or $mIgG_1$-FITC/Ta1-RD (2, 5, 8, 11) or CD23-FITC/Ta1-RD (3, 6, 9, 12) and then examined in flow cytometry analysis. The *x*-axis shows log green fluorescence and the *y*-axis shows red fluorescence. Quadrants delineated by squares indicate negative and positive populations of cells as determined using negative controls (1 and 2, 4 and 5, 7 and 8, 10 and 11)

*© Cancer Research Campaign 1999*

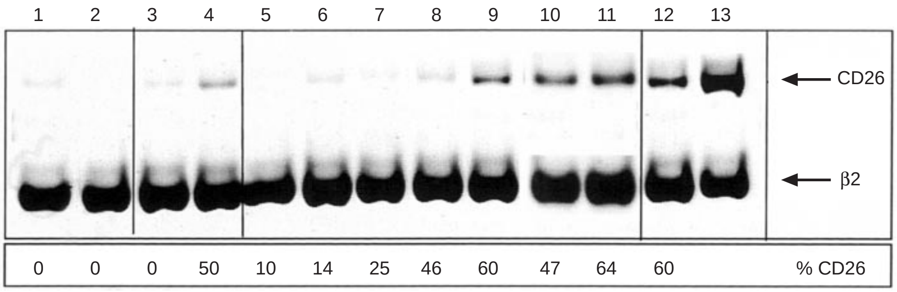

**Figure 4** Semi-quantitative PCR analysis of CD26 transcripts in normal and B-CLL cells. cDNAs from various B-cell populations were used as templates for PCR reactions using specific primers for CD26 or β2-microglobulin. PCR products were run on 7% acrylamide gels followed by ethidium bromide staining. Lanes 1–2, resting B-cells; lanes 3–4, day-2 B-cells untreated (lane 3) or treated with IL-4/SAC (lane 4); lanes 5–11, B-CLL cells from seven patients; lane 12, PHA-activated T-cells; lane 13, human dermal fibroblasts. Results of FACS analysis of cell surface CD26 expression were in parallel shown

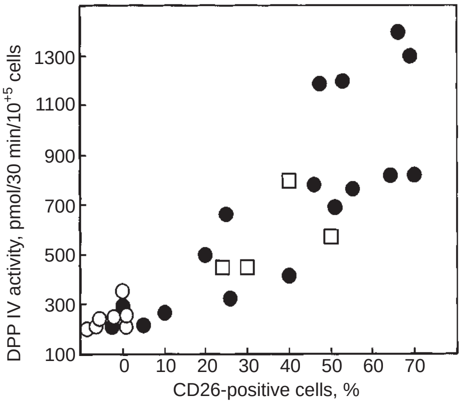

**Figure 5** Coanalysis of CD26 expression and DPP IV activity on B-CLL cells and normal counterpart B-cells. Cells were assessed for CD26 expression using Ta1 mAb and DPP IV activity using 1 mg ml$^{-1}$ Gly-Pro-pNA as substrate. B-CLL ● (*n* = 17); normal resting B-cells ○ (*n* = 7); normal B-cells activated in vitro with rIL-4/SAC □ (*n* = 4). CD26 expression was determined as the per cent of CD26-positive cells the possibility that soluble DPP IV activity originates from leukaemic B-cells is now under investigation.

In conclusion, the present study shows that B-CLL is associated with abnormal expression of plasma membrane CD26/DPP IV and suggests that this novel marker may have additional value in monitoring patients with other B-cell malignancies.

### ACKNOWLEDGEMENTS

The skillfull technical assistance in PCR of Mrs N Romquin was greatly appreciated. We thank Mrs S Chevillard for her advice in the choice of CD26 primers. We are grateful to Prof. F Sigaux (Hôpital Saint Louis, Paris) for the referral of some patients recruited to this study, and to Dr S Ezine for her dedicated helpfulness in FACS. We acknowledge Dr J Wietzerbin for advice and continual support. This work was supported by grants from Institut National de la Santé et de la Recherche Médicale (INSERM), the Association pour la Recherche sur le Cancer (ARC, n°1047) and Le Comité des Hauts-de-Seine de La Ligue Nationale Contre le Cancer.

### REFERENCES

Abbott CA, Baker E, Sutherland GR and McCaughan GW (1994) Genomic organization, exact localization, and tissue expression of the human CD26 (dipeptidyl peptidase IV) gene. *Immunogenetics* **40**: 331–338

Andrews C, Crockard AD, San Miguel JF and Catovsky D (1985) Dipeptidylaminopeptidase IV (DAPIV) in B- and T-leukemias. *Clin Lab Haematol* **7**: 359–368

Bauvois B (1997) Molecular and functional aspects of human myelomonocytic ectopeptidases. In *Cell Surface Peptidases in Health and Disease*, Kenny AJ and Boustead CM (eds), pp. 341–350. Bios Ltd: Oxford

Bauvois B, Sancéau J and Wietzerbin J (1992) Human U937 cell surface peptidase activities: characterization and degradative effect on tumour necrosis factor-α. *Eur J Immunol* **22**: 923–930

Bauvois B, Van Weyenbergh J, Rouillard D and Wietzerbin J (1996) TGF-β1-stimulated adhesion of human mononuclear phagocytes to fibronectin and laminin is abolished by IFN-γ: dependence on α5β1 and β2 integrins. *Exp Cell Res* **222**: 209–217

Bühling F, Junker U, Reinhold D, Neubert K, Jäger L and Ansorge S (1994) Functional role of CD26 on human B lymphocytes. *Immunol Lett* **45**: 47–51

Bylinkina VS, Lokshina LA, Samojlova RS, Lubkova ON, Gureeva TA and Golubeva NV (1992) Dipeptidylpeptidase IV in human leukemic B- and T-cells. *Biochem Int* **28**: 31–39

Carbone A, Cozzi M, Gloghini A and Pinto A (1994) CD26/dipeptidylpeptidase IV expression in human lymphomas is restricted to CD30-positive anaplastic large cells and a subset of T-cell non Hodgkin's lymphomas. *Hum Pathol* **25**: 1360–1365

Chomeczynski P and Sacchi N (1987) Single-step method of RNA isolation by acid guanidinium thiocyanate-phenol-chloroform extraction. *Anal Biochem* **162**: 156–159

De Meester I, Scharpé S, Vanham G, Bosmans E, Heylingen H, Vanhoof G and Corte G (1993) Antibody binding profile of purified and cell-bound CD26. Designation of BT5/9 and TA5.9 to the CD26 cluster. *Immunobiology* **188**: 145–158

De Meester I, Korom S, Belyaev A, Stadlbauer T, Goossens F, Haemers A, Kupiec-Weglinski JW and Scharpé S (1997) Inhibition of DPPIV/CD26 enzymatic activity abrogates accelerated rejection of rat cardiac allografts. *Immunol Lett* **56**: 487–491

Duke-Cohan JS, Morimoto C, Rocker JA and Schlossman SF (1996) Serum high molecular weight dipeptidyl peptidase IV (CD26) is similar to a novel antigen DPPT-L released from activated T cells. *J Immunol* **156**: 1714–1721

Eguchi K, Ueki Y, Shimomura C, Otsubo T, Nakao H, Migita K, Kawakami A, Matsunaga M, Tezuka H, Ishikawaw N, Ito K and Nagataki S (1989) Increment in the Ta1$^{+}$ cells in the peripheral blood and thyroid tissue of patients with Grave's disease. *J Immunol* **142**: 4233–4240

Feller AC, Parwaresch R and Lennert K (1983) Subtyping of chronic lymphocytic leukemia of T-type by dipeptidylpeptidase IV (DAP IV), monoclonal antibodies and Fc-receptors. *Cancer* **52**: 1609–1612

Fleisher B (1994) CD26: a surface protease involved in T-cell activation. *Immunol Today* **15**: 180–184

Flentke GR, Munoz E, Huber TB, Plaut AG, Kettner CA and Bachovchin WW (1991) Inhibition of dipeptidyl aminopeptidase IV (DP-IV) by Xaa-boroPro dipeptides and use of these inhibitors to examine the role of DP-IV in T-cell function. *Proc Natl Acad Sci USA* **88**: 1556–1559

*© Cancer Research Campaign 1999* 

Hafler DA, Fox DA, Manning ME, Schlossman SF, Reinherz EL and Weiner HL (1985) In vivo activated T lymphocytes in the peripheral blood and cerebrospinal fluid of patients with multiple sclerosis. *New Engl J Med* **312**: 1405–14011

Hegen M, Kameoka J, Dong RP, Schlossman SF and Morimoto C (1997) Cross-linking of CD26 by antibody induces tyrosine phosphorylation and activation of mitogen-activated protein kinase. *Immunology* **90**: 257–264

Invernizzi R, Bertolino G, Girino M, Perseghin P, Michiensi M and Nano R (1985) Cytochemistry of dipeptidylpeptidase IV and II in normal and neoplastic lymphoid cells. *Blut* **50**: 277–283

Jacotot E, Callebaut C, Blanco J, Krust B, Neubert K, Barth A and Hovanessian A (1996) Dipeptidylpeptidase IV-β, a novel form of cell surface expressed protein with dipeptidyl-peptidase IV activity. *Eur J Biochem* **239**: 248–258

Khalaf MR, Aqel NM and Hayhoe FGJ (1987) Histochemistry of dipeptidylaminopeptidase (DAP) II and IV in reactive lymphoid tissues and malignant lymphoma. *J Clin Pathol* **40**: 480–485

Kolb JP, Genot E, Poggioli J, Abadie A, Sarfati M, Delespesse G and Dugas B (1991) Transduction through CD23: different signalling pathways in human B cells and monocytes. *Monogr Allergy* **29**: 135–141

Kondo S, Kotani T, Tamura K, Aratake Y, Uno H, Tsubuchi H, Inoue S, Niho Y and Ohtaki S (1996) Expression of CD26/dipeptidylpeptidase IV in adult T cell leukemia/lymphoma (ATLL). *Leuk Res* **20**: 357–363

Letarte M, Greaves A and Ishii E (1997) The biological functions of CD10/endopeptidase 24.11 in normal and malignant cells. In *Cell Surface Peptidases in Health and Disease*, Kenny AJ and Boustead CM (eds), pp. 329–337. Bios Ltd: Oxford

Nakao H, Eguchi K, Kawakami A and Nagataki S (1989) Increment of Ta1 positive cells in peripheral blood from patients with rheumatoid arthritis. *J Rheumat* **16**: 7–10

Schön E, Jahn S, Kiessig T, Demuth HU, Neubert K, Barth A, Von Baehr R and Ansörge S (1987) The role of dipeptidylpeptidase IV in human T lymphocyte activation: inhibitors and antibodies against dipeptidylpeptidase IV suppress lymphocyte proliferation and immunoglobulin synthesis in vitro. *Eur J Immunol* **17**: 1821–1826

Scott CS, Stark AN, Minowada J and Drexler HG (1988) Quantitative and qualitative studies of leukemic cell dipeptidyl peptidase II and IV. *Leuk Res* **12**: 129–134

Srivastava MD and Bhargava AK (1986) Expression of dipeptidylaminopeptidase-IV in some human T and B cell lines. *Acta Haematol* **76**: 25–28

Tanaka T, Umeki K, Yamamoto Y, Akamoto F, Noguchi and Ohtaki, S (1995) CD26 (dipeptidylpeptidase IV/DPP IV) as a novel molecular marker for differentiated thyroid carcinoma. *Int J Cancer* **64**: 326–331

Uematsu T, Urade M, Yamaoka M and Yoshioha M (1996) Reduced expression of dipeptidylpeptidase (DPP) IV in peripheral blood T lymphocytes of oral cancer patients. *J Oral Pathol Med* **25**: 507–512

Vanham G, Kestens L, De Meester I, Vingerhoets J, Penne G, Vanhoof G, Scharpé S, Heyligen H, Bosmans E, Ceuppens JL and Gigase (1993) Decreased expression of the memory marker CD26 on both CD4+ and CD8+ T lymphocytes of HIV-infected subjects. *J Acquir Immune Defic Syndr* **6**: 749–757

Yokotani N, Doi K, Wenthold J and Wada K (1993) Non-conservation of a catalytic residue in a dipeptidyl aminopeptidase IV-related protein encoded by a gene on human chromosome 7. *Hum Mol Genet* **2**: 1037–1039

*© Cancer Research Campaign 1999*
